# The prognostic significance of protein arginine methyltransferase 6 expression in colon cancer

**DOI:** 10.18632/oncotarget.23809

**Published:** 2017-12-27

**Authors:** Yongchul Lim, Suyeun Yu, Jung-A Yun, In-Gu Do, Lan Cho, Yang Hee Kim, Hee Cheol Kim

**Affiliations:** ^1^ Department of Surgery, Samsung Medical Center, Sungkyunkwan University School of Medicine, Seoul, Korea; ^2^ Department of Preventive Medicine, College of Medicine, Korea University, Seoul, Korea; ^3^ Department of Surgery, Hanyang University Guri Hospital, Hanyang University College of Medicine, Guri, Korea; ^4^ Department of Pathology, Kangbuk Samsung Hospital, Sungkyunkwan University School of Medicine, Seoul, Korea; ^5^ Department of Surgery, Samsung Medical Center, Biomedical Institute, Seoul, Korea

**Keywords:** colon cancer, protein arginine methylation, PRMT6, apoptosis, prognosis

## Abstract

Protein arginine methylation is involved in cellular differentiation and proliferation. Recently, aberrant expression of protein arginine methyltransferases, which are responsible for the methylation reaction, has been reported in various types of cancer. However, there is no clear evidence regarding the prognostic value of abnormal PRMT6 expression in colorectal cancer or the effect of PRMT6 regulation on CRC cells. We investigated the expression patterns of PRMT6 in patients with stage II and III CRC. We detected nuclear expression of PRMT6 in 23.7% of carcinoma samples by immunohistochemistry. Among the clinicopathological parameters, the ratio of poorly differentiated cancer cells was approximately two-fold higher in patients with PRMT6-positive disease than in those with PRMT6-negative disease (*p* = 0.002). Patients with PRMT6-positive CRC had a shorter disease-free survival than those with PRMT6-negative CRC in both univariate and multivariate analyses (*p* = 0.018 and *p* = 0.035, respectively). siRNA-mediated inhibition of PRMT6 expression in CRC cells induced p21^WAF1/CIP1^ overexpression and suppressed cell growth and colony-forming ability. Concomitantly, apoptosis was induced in PRMT6-suppressed CRC cells. These data suggest that PRMT6 can serve as a biomarker for unfavorable prognosis and as a therapeutic target in CRC.

## INTRODUCTION

Colorectal cancer (CRC) is one of the most commonly diagnosed cancers and fourth most common cause of cancer-related death worldwide [1]. CRC typically develops slowly, over a period of 10–20 years [2], and the accumulation of genetic and epigenetic alterations is involved in the initiation and progression of the disease. The most notable genetic changes in colorectal carcinogenesis include alterations in *APC*, *KRAS*, *SMAD4*, *TP53*, and the mismatch repair genes *MLH1* and *MSH2* [3, 4]. Hypermethylation of CpG islands in a certain panel of genes also contributes to disease development, and this phenotype is often associated with *BRAF* mutations [5]. Nevertheless, the molecular pathogenesis of CRC is heterogeneous and remains poorly understood.

Arginine methylation is a common posttranslational modification catalyzed by protein arginine methyltransferases (PRMTs) that use *S*-adenosylmethionine as the methyl donor [6]. PRMTs are classified into two groups according to their products. Type I enzymes (PRMT1, 2, 3, 4, 6, and 8) can generate asymmetric *N*G, *N*G-dimethyl-arginine, while type II (PRMT5 and PRMT7) produce symmetric *N*G, *N′*G-dimethyl-arginine. Arginine methylation has been implicated in signal transduction, transcription, mRNA splicing, and DNA damage response, and affects protein-protein interactions and protein localization [7]. Correspondingly, arginine methylation has been linked to carcinogenesis, metastasis, and drug resistance [8, 9], and dysregulation of PRMTs is often associated with diverse types of cancer [10]. Recently, some PRMTs have emerged as promising targets for cancer therapeutic strategies [11].

Among the PRMTs, PRMT6 is localized exclusively in the nucleus [12] and the enzyme has been implicated in the regulation of nuclear processes, such as DNA repair and gene expression [13, 14]. Previously, we reported that PRMT6 expression is gradually reduced during the replicative senescence of WI-38 fibroblasts [15] and increased when the cell cycle proceeds from G0/G1 to S phase in HeLa cells [16]. It has been also reported that mouse embryonic fibroblasts from PRMT6^−/−^ embryos undergo rapid cellular senescence [17] and depletion of PRMT6 in MCF7 breast cancer cells induces senescence as well as cell cycle arrest [18]. The mechanism responsible for these phenomena is that PRMT6 acts as a transcriptional co-repressor by directly binding to the promoters of tumor suppressor genes such as p21 (*CDKN1A* or p21 ^waf1/CIP1^) and p53 (TP53), where it methylates histone H3 arginine 2 [17–19]. In addition, methylation of p21 at arginine 156 by PRMT6 promotes the phosphorylation of threonine 145 on the protein and increased cytoplasmic localization of p21, resulting in HCT116 cell resistance to doxorubicin [20].

In clinical studies, PRMT6 was found to be upregulated in breast, cervix, bladder, prostate, and lung cancers [21, 22]. However, the expression level of PRMT6 in CRC tissues and effect of the enzyme on CRC cell proliferation remain unclear. Therefore, we investigated whether PRMT6 positivity in clinical samples is significantly associated with clinicopathophysiologic features and patient survival. In addition, we examined whether PRMT6 depletion affects CRC cell proliferation and apoptosis.

## RESULTS

### PRMT6 is overexpressed in CRC tissues

**Figure 1:**
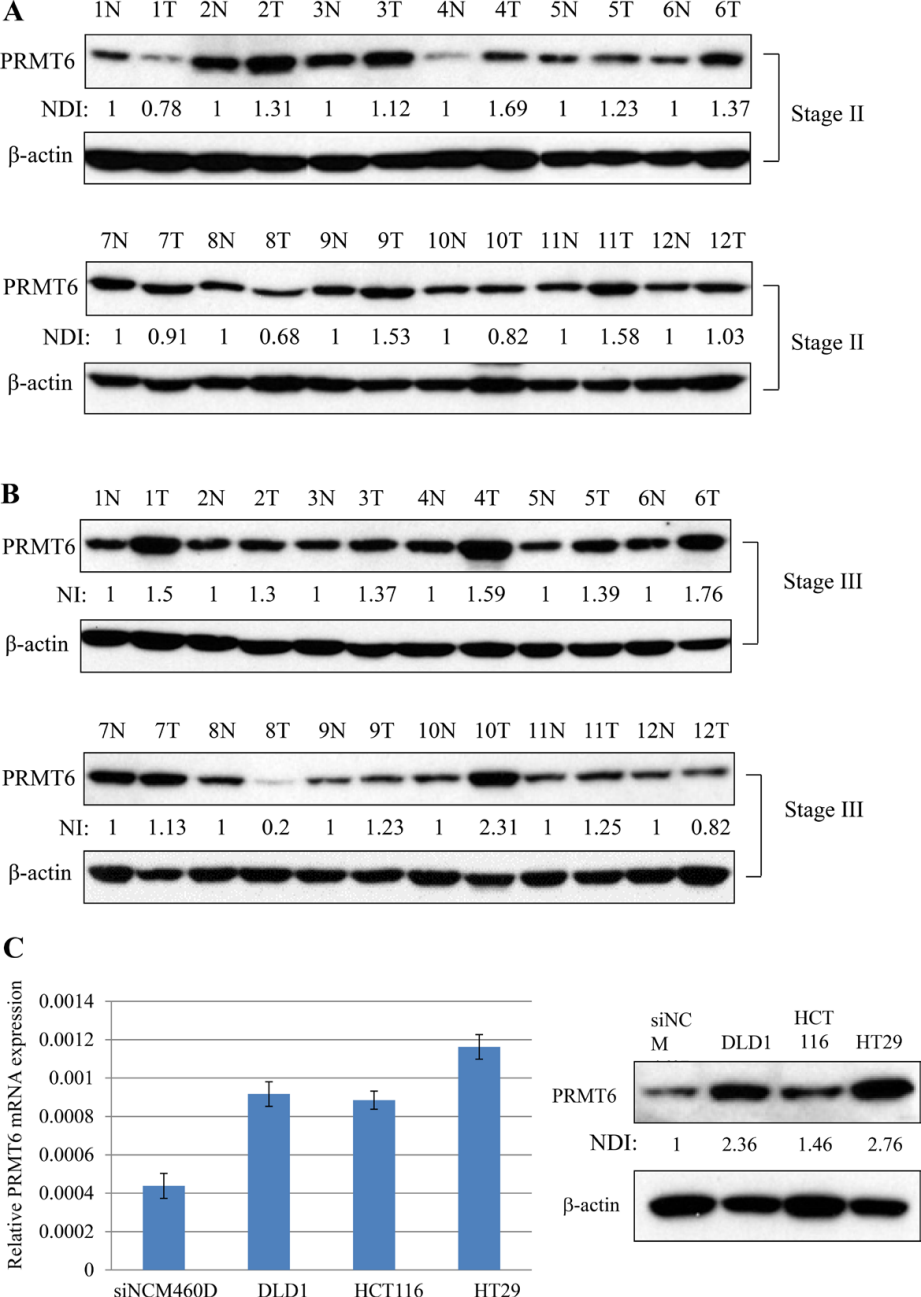
Overexpression of PRMT6 in CRC tissues and cell lines (**A**) Comparison of PRMT6 expression between the primary CRC and adjacent normal tissues paired from the same patient with stage II. (**B**) The same experiments in panel (A) were carried out with tissue extracts from the patients with stage III. (**C**) PRMT6 mRNA and protein expression levels between NCM460D and three CRC cells were compared by real-time PCR (left panel) and western blotting (right panel), respectively. Densitometric intensity of PRMT6 protein was normalized to β-actin. NDI indicates normalized densitometric intensity.

We compared the expression level of PRMT6 in 24 matched samples from cancer tissues and adjacent normal tissues from the same patients by western blotting. As shown in Figure 1A and 1B, PRMT6 expression increased by more than 1.5-fold in seven tumor samples matched with adjacent noncancerous tissues from the same patients. In addition, comparison of PRMT6 mRNA and protein levels between normal epithelial cells (NCM460D) derived from human colon mucosa and three CRC cell lines (DLD1, HCT116, and HT29) revealed that the three CRC lines highly expressed the PRMT6 gene compared to the NCM460D cells (Figure 1C). Among the CRC cell lines, both the mRNA and protein levels of PRMT6 were highest in HT29 cells (Figure 1C).

### PRMT6 expression is associated with clinicopathologic characteristics of CRC patients

**Figure 2:**
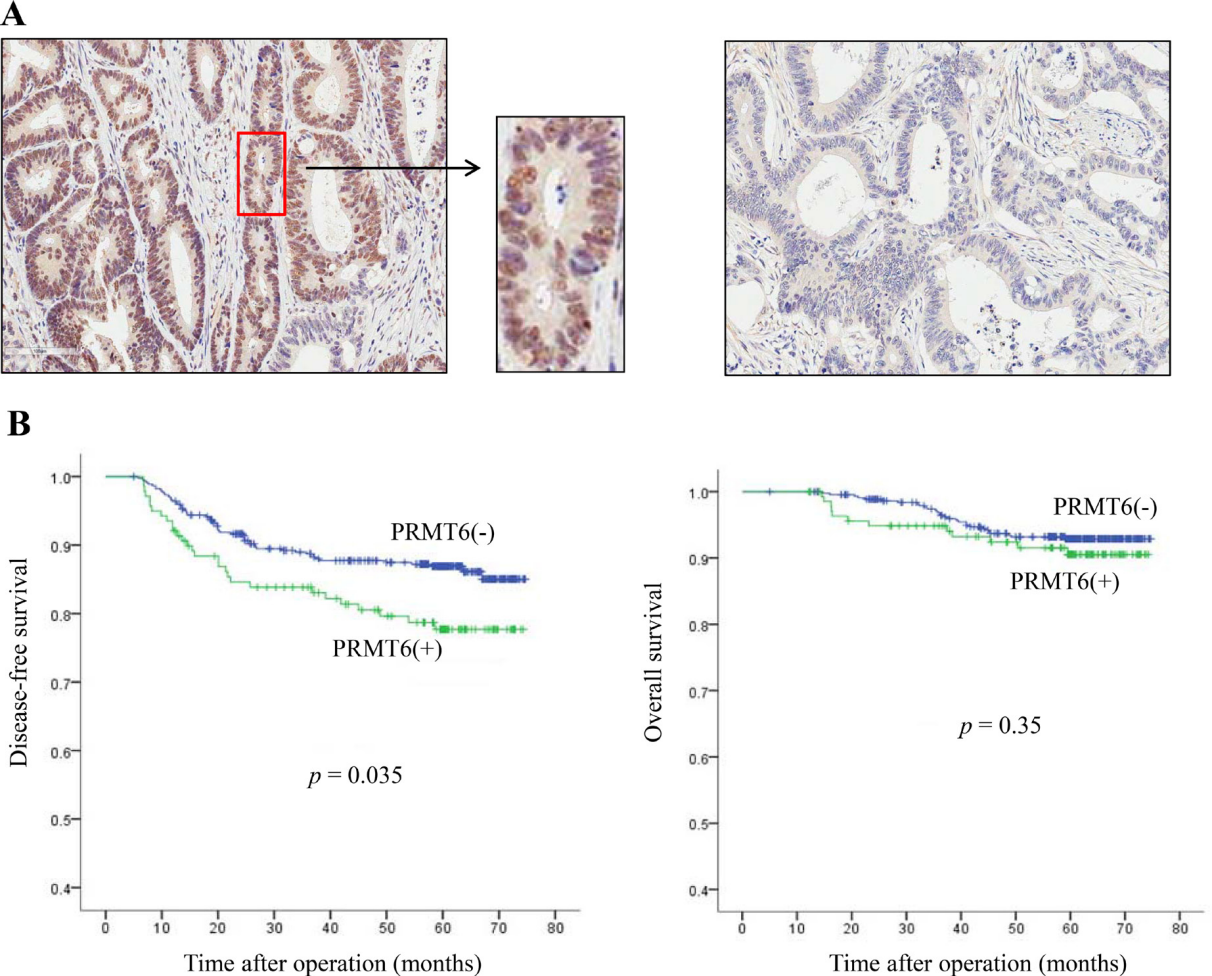
Clinical significance of PRMT6 overexpression in CRC tissues (**A**) Representative images (× 100) of PRMT6 positive (left panel) and PRMT6-negative tumors (right panel) stained with anti-PRMT6 antibody from stage III CRC patients. (**B**) Kaplan-Meier survival curves according to the immunoexpression of PRMT6 protein. The left and right panels show disease-free and overall survival, respectively.

To determine the clinicopathological associations of PRMT6 in patients with CRC, we performed immunohistochemistry on 586 CRC tissue samples. We found positive staining for PRMT6 in 23.7% of CRC samples (139 of 586 patients) (Table 1 and Supplementary Table 2), and the enzyme was localized exclusively in the nucleus (Figure 2A and Supplementary Figure 1). Statistical analysis of PRMT6 positivity and standard clinicopathological variables showed that the ratio of poorly differentiated, mucinous, and signet ring cells in the cell type was approximately two-fold higher in the group with PRMT6 expression (15.8%) than in the group without PRMT6 expression (7.2%) (*p* = 0.002), indicating that PRMT6 activity plays a critical role in cancer cell differentiation (Table 1). However, PRMT6 positivity did not show a significant association with other standard clinicopathological parameters (Table 1).

**Table 1:** The association of the expression of PRMT6 (negative/positive) with the clinicopathologic variables

	PRMT6		*p*-value
	Negative (*N* = 447)	Positive (*N* = 139)	
**Gender**			
**Female**	169 (37.8)	52 (37.4)	0.933
**Male**	278 (62.2)	87 (62.6)	
**Location**			
**Colon**	327 (73.2)	109 (78.4)	0.214
**Rectum**	120 (26.8)	30 (21.6)	
**CEA level**			
**≤ 5 ng/ml**	362 (81.0)	116 (83.5)	0.512
**> 5 ng/ml**	85 (19.0)	23 (16.5)	
**T stage**			
**T1-2**	35 (7.8)	5 (3.6)	0.084
**T3-4**	412 (92.2)	134 (96.4)	
**N stage**			
**N0**	230 (51.5)	78 (56.1)	0.336
**N+**	217 (48.5)	61 (43.9)	
**Cell type**			
**WD/MD**	415 (92.8)	117 (84.2)	0.002
**PD/Muc/SRC**	32 (7.2)	22 (15.8)	
**Lymphatic invasion**			
**Negative**	328 (73.4)	102 (73.4)	0.999
**Positive**	119 (26.6)	37 (26.6)	
**Vascular invasion**			
**Negative**	390 (87.2)	114 (82.0)	0.120
**Positive**	57 (12.8)	25 (18.0)	
**Perineural invasion**			
**Negative**	413 (92.4)	130 (93.5)	0.655
**Positive**	34 (7.6)	9 (6.5)	
**Adjuvant chemotherapy**			
**Yes**	353 (79.0)	105 (75.5)	0.637
**No**	34 (7.6)	11 (7.9)	
**Undescribed**	60 (13.4)	23 (16.5)	

Next, to evaluate the correlation between PRMT6 expression and overall survival (OS) and disease-free survival (DFS), we first performed univariate analysis of traditional clinicopathological variables for prognosis. The presence of PRMT6 showed significance (*p = 0.018*) in the analysis of DFS with other clinicopathological factors (Supplementary Table 3). In multivariate analysis, PRMT6 positivity was also found to be an independent prognostic factor for shorter DFS (*p = 0.035*, Supplementary Table 3). Kaplan-Meier curves depicted significant differences in DFS between patients whose tumors had positive and negative expression of PRMT6 (*p* = 0.035), but not in OS (*p* = 0.350) (Figure 2B). These results indicate that PRMT6 expression is a useful indicator of unfavorable prognosis in patients with CRC.

### Knockdown (KD) of PRMT6 inhibits growth and colony-formation in CRC cell lines

**Figure 3:**
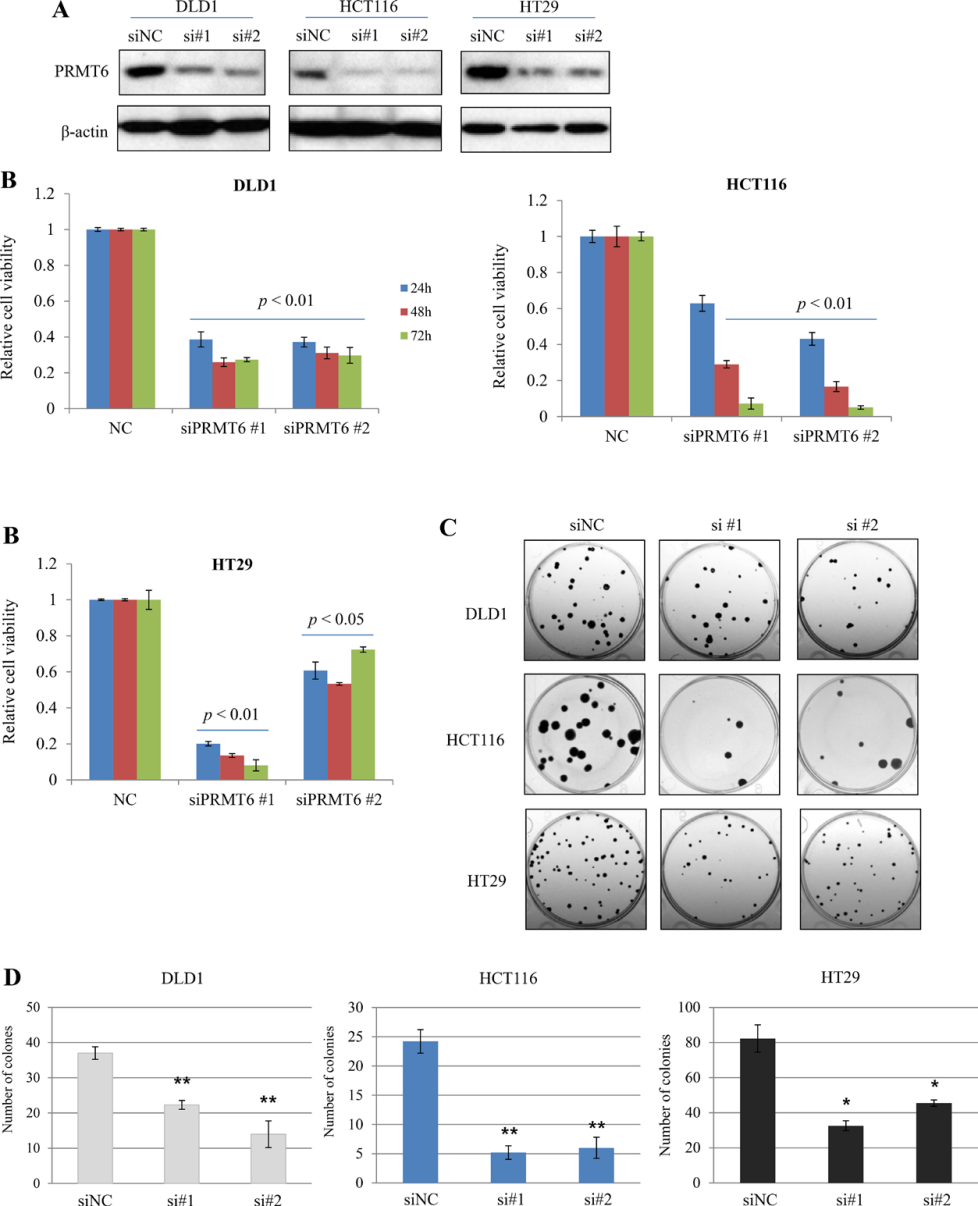
Inhibition of growth and colony-forming ability by downregulation of PRMT6 in CRC cell lines (**A**) Knockdown of endogenous PRMT6 in three CRC cell lines by siRNAs transfection. (**B**) Suppression of PRMT6 inhibits cell growth as determined by a WST-1 based assay. (**C**) Representative stained plates of the CRC cells are shown and (**D**) the number of colonies was evaluated. Error bars denote SEM from three independent experiments. ^*^, *p* < 0.05; ^**^, *p* < 0.01.

To investigate the importance of PRMT6 in the proliferation of CRC cells, two different siRNA duplexes against human PRMT6 (siPRMT6 #1, #2) were used to knockdown the enzyme in three human CRC cell lines. Three days after siRNAs transfection, we examined cell proliferation activity. Two siRNAs for PRMT6 efficiently suppressed the expression of endogenous PRMT6 protein at 72 h post-transfection (Figure 3A) and PRMT6 KD significantly inhibited proliferation of CRC cells compared to in negative control siRNA (siNC)-transfected cells (Figure 3B). We also compared the colony-forming capability between siNC- and siPRMT6-transfected CRC cells. PRMT6-suppressed cells showed a marked reduction in colony formation rates compared with siNC-transfected cells (Figure 3C and 3D). These findings suggest a role for PRMT6 in promoting CRC cell proliferation and in the progression of CRC.

### Knockdown of PRMT6 upregulates tumor suppressor p21 protein in CRC cells

**Figure 4:**
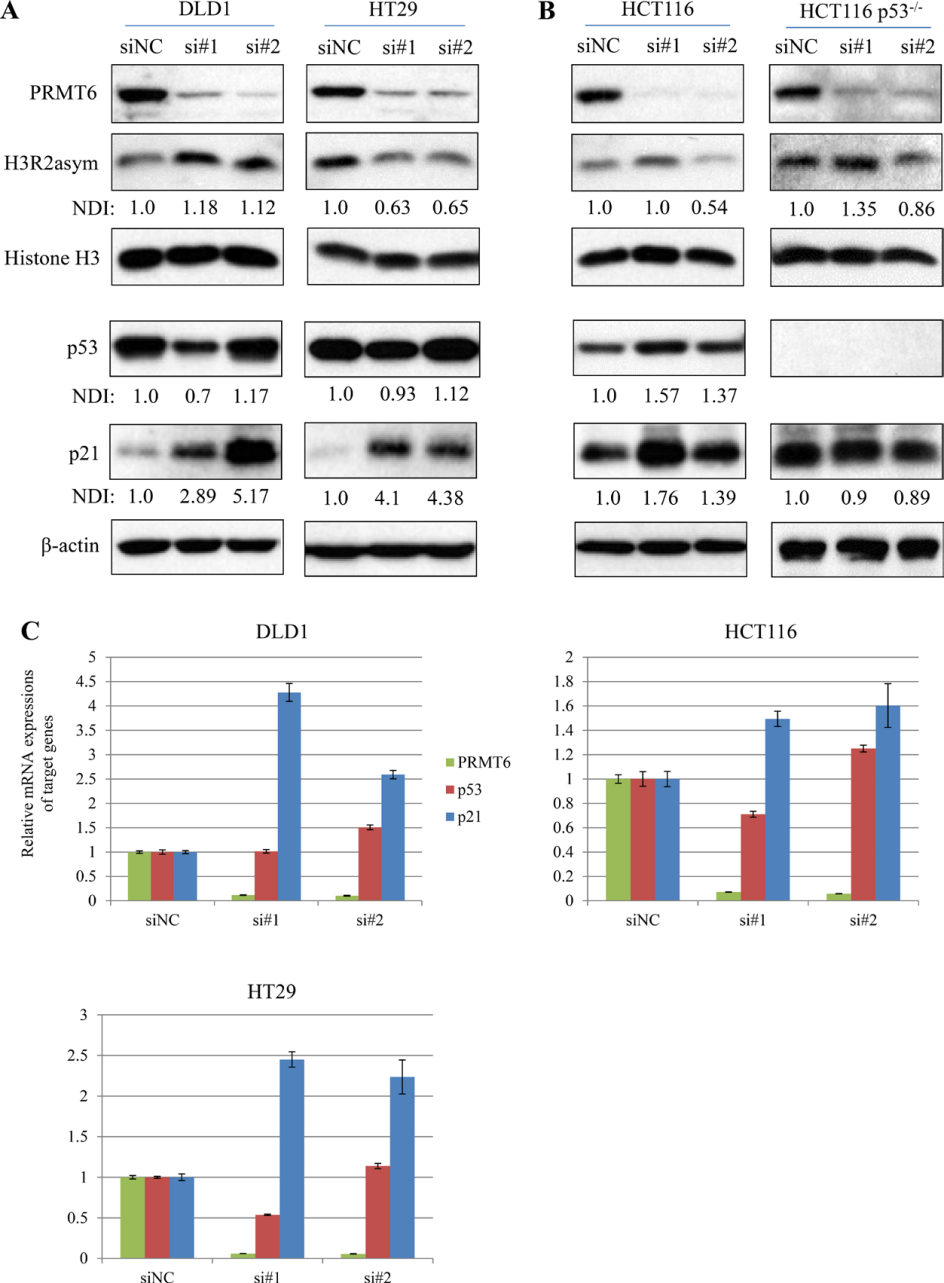
Changes in histone H3R2me2a and tumor suppressors expression in PRMT6-knockdown cells (**A**) Total cell extracts were prepared from siNC- and siPRMT6s transfected DLD1 and HT29 cells. Western immunoblots were carried out with the indicated protein antibodies. Densitometric intensity of each protein was normalized to b-actin. NDI indicates normalized densitometric intensity. (**B**) Using HCT116 p53^+/+^ and HCT116 p53^−/−^ cell extracts, the same experiments in panel (A) were carried out. (**C**) mRNA expression of p53, p21, and PRMT6 was compared between siNC- and siPRMT6-transfected CRC cells.

In previous studies, PRMT6 was shown to function as a transcriptional repressor and control cell proliferation by H3R2 dimethylation and subsequent repression of tumor suppressors such as p21 and p53 [17–19]. Based on these reports, we examined the inter-relationships between PRMT6 and two tumor suppressor expressions as well as levels of H3R2 methylation in three CRC cells. CRC cell lines transfected with each of two siPRMT6s commonly showed significant increases in both p21 protein (Figure 4A and 4B) and mRNA (Figure 4C). However, the changes in p53 expression and H3R2 dimethylation status were cell-type specific. DLD1 showed neither p53 induction nor H3R2 hypomethylation under PRMT6 knockdown, while PRMT6 downregulation in HT29 cells induced significant reductions in H3R2 methylation without altering p53 levels compared to siNC-transfected cells (Figure 4A). In HCT116 cells, PRMT6 knockdown induced p53 and p21 expression without H3R2 hypomethylation (Figure 4B left panel). To investigate the importance of p53 in siPRMT6-mediated p21 induction in HCT116 cells, each siPRMT6 was transfected to a p53-null isogenic cell line. Although PRMT6 levels were significantly reduced in siPRMT6-transfected isogenic cells, p21 expression was not induced (Figure 4B right panel), indicating that p21 induction by PRMT6 depletion depends on p53 upregulation in HCT116 cells. These results indicate that regulation of p21 expression involving PRMT6 is complex and depends on the cellular context and circumstances.

Next, we investigated the correlation between PRMT6 and p21 proteins as well as p53 in 24 tumor tissue samples as shown in Figure 1A by western blotting. When densitometric values corresponding to the two tumor suppressors and PRMT6 proteins were plotted and analyzed, an inverse correlation as shown in CRC cells was not observed in clinical samples from CRC patients (Supplementary Figure 2).

### PRMT6 knockdown facilitates apoptosis of CRC cell lines

**Figure 5:**
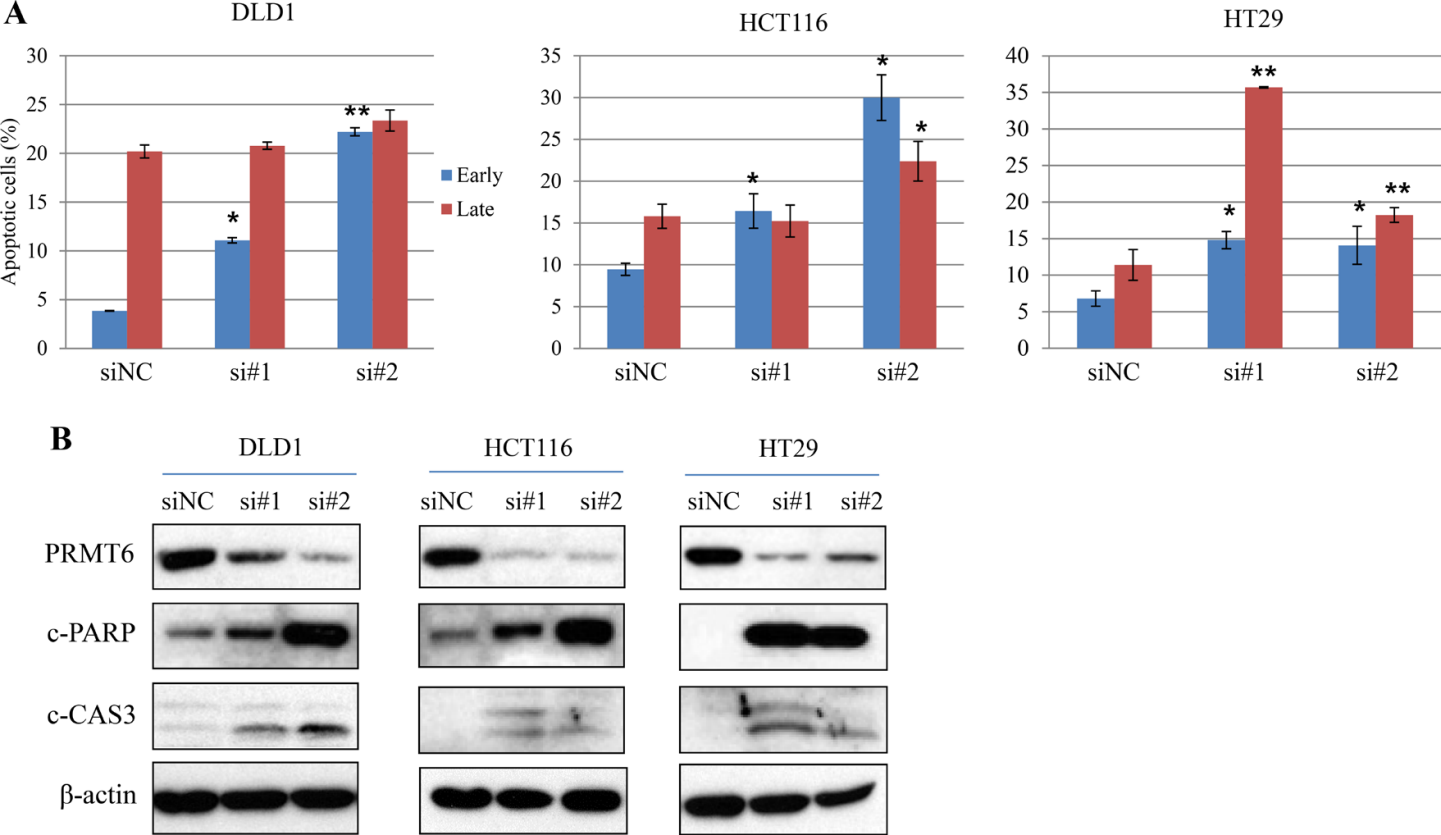
Induction of apoptosis of three CRC cells by PRMT6 knockdown (**A**) Quantification of the mean annexin-V and propidium iodide fluorescence in early and late apoptotic cell populations with three independent experiments. Representative scatter plots of each cell lines are presented in Supplementary Figure 3. ^*^, *p* < 0.05; ^**^, *p* < 0.01. (**B**) Induction of cleaved forms of caspase 3 and PARP proteins in PRMT6-suppressed CRC cells.

To further investigate the effect of PRMT6 on apoptosis in CRC cells, we compared the ratio of apoptotic cells between siNC- and siPRMT6-transfected cells by flow cytometry. As shown in Figure 5A and Supplementary Figure 3, inhibition of PRMT6 expression resulted in significant induction of apoptosis in all CRC cells tested. To confirm these results, the expression levels of apoptosis-related proteins were assessed, and we found that both the active form of caspase 3 and PARP degradation were clearly increased by PRMT6 depletion in three CRC cell lines (Figure 5B). Collectively, these results demonstrate that PRMT6 overexpression plays a critical role in suppressing apoptosis in CRC cells and that the enzyme can serve as an effective therapeutic target in CRC.

## DISCUSSION

Fundamental biological processes regulated by protein arginine methylation include RNA processing, protein trafficking, signal transduction, and transcription [23]. Particularly, recent experimental evidence regarding genomic instability, DNA repair, and metastatic potential indicated oncogenic pathways involving arginine modification [8].

In the present study, we found that 23.7% of colorectal cancers showed positive staining of PRMT6 by immunohistochemistry and that the localization of the enzyme was exclusively nuclear. Among the clinical and pathological parameters listed in Table 1, PRMT6 expression was significantly associated with the degree of cancer cell differentiation. Although PRMT6 expression was not related to the OS of patients with CRC, Cox univariate and multivariate analyses revealed significant relationships between PRMT6 positivity and shorter DFS. These results were confirmed by Kaplan-Meier analysis. Previously, the expression of specific isoforms of PRMT1 variants were shown to be significantly associated with nodal status, TNM stage, and tumor grade [24, 25], and both PRMT4 and PRMT5 are highly expressed in colon cancer [26, 27]. However, among the PRMTs tested, only PRMT6 showed a significant association with disease-free survival, indicating that enzyme expression is a useful indicator of unfavorable prognosis for colon cancer.

Tumor suppressors such as p53, *CDKN1B* (p27), and p21 are often silenced or inactivated in human cancers, thereby allowing deregulation of CDK activity and cell cycle progression. In CRC, however, the expression of these genes as prognostic markers remains unclear [28, 29]. In addition, although p21 protein serves as a surrogate indicator for p53 protein alterations, there are discrepancies between p53 and p21 expression levels in CRC [30]. Collectively, these findings indicate the presence of p53-independent mechanisms of p21 regulation during CRC development. In the present study, we found that siRNA-mediated PRMT6 depletion significantly induced p21 expression in all the three CRC cell lines (Figures 4A–4C). Based on these results and those of previous studies [17–19], we examined the inverse correlation between hypomethylation of H3R2 by PRMT6 suppression and p21 induction in the three CRC cells. However, a correlation was only observed for HT29 cells (Figure 4A). For p53, while DLD1 and HT29 cells carrying mutated p53 (S241F and R273H, respectively [31]) showed no induction of p53 under PRMT6 suppression, HCT116 cells with wild-type p53 showed p53 upregulation (Figure 4A and 4B, respectively). Neault et al. [17]. reported that PRMT6 negatively regulates p53 gene expression by methylating H3R2 and consequently p21 expression is repressed in mouse embryonic fibroblasts. However, hypomethylation of H3R2 in PRMT6-suppressed HCT116 cells was not induced (Figure 4B). We analyzed the correlation between the two tumor suppressors and PRMT6 expression in clinical tissue samples as shown in Figure 1A; however, no inverse correlation shown in CRC cell lines was observed in CRC tissues (Supplementary Figure 2). These findings suggest that p21 and p53 regulation by PRMT6 is cell type-specific rather than a general phenomenon in CRC.

Nakakido et al. [20] reported that arginine residue 156 in the p21 protein is methylated by PRMT6, enhancing cytosolic localization of the protein in HCT116 cells. We also detected p21 and PRMT6 in both the nuclear and cytosolic fractions, with much higher levels in the cytoplasm (Supplementary Figure 4A). Moreover, when PRMT6 expression was suppressed in HCT116 cells, the p21 ratio in the nucleus to cytoplasm increased compared to that of siNC (Supplementary Figure 4B). These results are consistent with those of Nakakido et al. In contrast, DLD1 and HT29 cells did not show an increased ratio of p21 in the nucleus to cytoplasm under PRMT6-KD (Supplementary Figure 4B), suggesting that the regulation of p21 localization by PRMT6 is not a general phenomenon in CRC cells.

PRMT6 suppression induced apoptosis in CRC cells and increased the expression of the cleavage forms of caspase 3 and PARP (Figure 5). As described above, PRMT6 depletion induces cell cycle arrest and senescence in several types of cancer cell lines [17,19,20]; however, PRMT6-mediated apoptosis in cancer cells has not been previously reported. Our results showed that PRMT6 is a promising target for pharmaceutical drug development, and the molecular mechanism underlying PRMT6-mediated CRC cell apoptosis should be further investigated.

## MATERIALS AND METHODS

### Patient samples

From June 2008 to May 2009, 1035 patients diagnosed with primary CRC underwent radical surgery at the Samsung Medical Center (Sungkyunkwan University School of Medicine) in Seoul, Korea. We obtained prior patient consent as well as approval from the Institutional Research Board to use clinical materials for research purposes. Among these patients, 621 were diagnosed with pathological stage II, IIIA, or IIIB CRC after surgery for the primary tumor. Following the exclusion of 26 patients with no tumors in specimens because they had already undergone previous polypectomy or endoscopic resection of the primary tumor before surgery and nine patients with damaged or unreadable slides, a total of 586 patients were included in the analysis. Comprehensive chart reviews were performed to obtain clinicopathological information, and follow-up data were obtained from the medical records and National Bureau of Statistics. The included variables were sex, age, tumor location (colon and rectum), preoperative carcinoembryonic antigen level (ng/mL), pathologic TNM stage, histopathological type (tumor differentiation status), lymphatic invasion, vascular invasion, perineural invasion, adjuvant chemotherapy, and expression of PRMT6 (negative/positive).

Adjuvant chemotherapy is typically recommended for patients diagnosed with stage III cancer or stage II cancer with high-risk factors. In our hospital, poor histopathological differentiation, positive lymphatic invasion, positive vascular invasion, and positive perineural invasion were regarded as high-risk factors in patients with stage II cancer. Adjuvant chemotherapy was performed from 2 to 3 weeks after discharge, and the most common treatment regimens were the following: (1) ftorafur plus uracil (*n* = 157); (2) capecitabine (Xeloda^®^) (*n* = 129); (3) oxaliplatin-based chemotherapy (*n* = 85); and (4) intravenous 5-fluorouracil-based chemotherapy (*n* = 12). Among patients with rectal cancers, 75 patients received pre- and post-operative radiotherapy.

### Preparation of colon tissue extracts

Twenty four frozen CRC tumors and matched adjacent normal tissues were collected after surgery and the tissue samples were immediately frozen in liquid nitrogen. For protein extraction, 50–100 mg of tissue was homogenized using a TissueLyser (Qiagen, Hilden, Germany) in lysis buffer (20 mM HEPES, pH 8.0, 9.0 M urea, 1× protease and phosphatase inhibitors) and sonicated with three bursts of 30 s at 15 W. The extracts were then centrifuged at 20,000 × *g* at 4°C for 15 min and the supernatant was stored at −80°C until use.

### Tissue microarray construction and immunohistochemical stains

Tissue microarrays were constructed using the Quick Ray^®^ Manual Tissue Microarrayer (Unitma Co., Ltd. Seoul, Korea). Specimens from four representative tumor regions were taken from donor formalin-fixed paraffin-embedded blocks using a 2-mm core punch and arrayed into recipient blocks. Four 1-μm-thick tissue microarray sections were labeled with rabbit polyclonal anti-PRMT6 antibody (Bethyl Laboratories, Inc., Montgomery, TX, USA). Tissue sections were deparaffinized three times in xylene for a total of 15 min and subsequently rehydrated. Immunostaining was performed using a Bond-Max autoimmunostainer with a Bond^TM^ Polymer Refine Detection kit, DS9800 (Leica Biosystems, Wetzlar, Germany). Briefly, antigen retrieval was carried out at 97°C for 20 min in Bond^TM^ Epitope Retrieval Solution 1. After blocking endogenous peroxidase activity with 3% hydrogen peroxidase for 10 min, primary antibody incubation was carried out for 15 min at room temperature at a dilution of 1:200. Counter-staining was performed with Mayer’s hematoxylin. Negative controls (substitution of TBS for primary antibody) were run simultaneously. Staining for PRMT6 was considered positive when tumor cells showed nuclear reactivity in more than two of four cores (Figure 2A).

### Cell culture

The human normal epithelial cell line (NCM460D) derived from the colon mucosa was purchased from INCELL Corporation (San Antonio, TX, USA). NCM460D cells were cultured in MA3 medium. Three human colon cancer cell lines, DLD-1, HCT116, and HT29, were obtained from the American Type Culture Collection (Manassas, VA, USA). DLD1 and HT29 cells were cultured in RPMI-1640. HCT116 cells and p53-null HCT116 cells were maintained in McCoy’s 5A medium. All culture media were supplemented with 10% fetal bovine serum and 1% penicillin/streptomycin (GIBCO, Grand Island, NY, USA). Cells were maintained at 37°C with 5% CO_2_.

### SiRNA transfection

SiRNA oligonucleotide duplexes were purchased from Integrated DNA Technologies (Coralville, IA, USA). The siRNA sequences were as follows: PRMT6 siRNA #1 (sense, ACAGCAUACCUAAGAAACUCAGAA G; antisense, CUUCUGAGUUUCUUAGGUAUGCUGUAC), PRMT6 siRNA #2 (sense, CUACUUACAAGUAGUGAAAGUUCCC; antisense, GGGAACUUUCACUACUUGUAAGUA GGC). Cells in six-well culture plates were transfected with increasing concentrations of siRNAs (see Figure 1) using Lipofectamine RNAiMAX (Life Technologies, Carlsbad, CA, USA) according to the manufacturer’s instructions.

### Cell proliferation assay

The metabolic activity of cells was assessed by indirectly measuring cell viability using CellVia (AbFrontier, Seoul, Korea) according to the manufacturer’s instructions. CRC cells were transfected with PRMT6 siRNAs as described above and seeded in 96-well plates at a density of 5 × 10^3^ cells in 100 μL medium per well. After transfection, at each point, 10 μL of the reagent was added to each well and the plates were incubated at 37°C for 3 h Reduction of the water-soluble tetrazolium salt WST-1 to formazan was determined using an enzyme-linked immunosorbent assay reader at 450 nm.

### Colony formation assay (CFA)

We seeded 100 viable cells per well into six-well plates 24 h post-transfection. The cells were cultured for 10–14 days with media changes every 2–3 days. To visualize the colonies, the medium was removed and the cells were washed with PBS and stained with a staining solution containing 0.5% crystal violet and 6% glutaraldehyde for 30 min at room temperature [32]. The colonies were scored under a microscope and data are presented as the mean number of colonies ± standard deviation (SD) from three independent experiments.

### Apoptosis assay

Apoptosis was quantitated by flow cytometry using an annexin V-fluorescein isothiocyanate (FITC)/propidium iodide (PI) kit according to the manufacturer’s instructions (BD Bioscience, Franklin Lakes, NJ, USA). After 48 h post-transfection with siNC and siPRMT6s, Cells were washed with PBS and suspended in annexin V binding buffer, and annexin V-FITC solution and PI were added. Next, the cells were incubated at room temperature for 15 min. The stained cells were analyzed by fluorescence-activated cell sorting (BD FACSAria™; BD Biosciences). The data were analyzed using FACSDiva™ software (BD Biosciences).

### Western blot analysis

Equal amounts of colon tissue or cancer cell extracts were subjected to sodium dodecyl sulfate polyacrylamide gel electrophoresis and transferred onto polyvinylidene fluoride membranes (Millipore, Billerica, MA, USA). The membranes were incubated overnight at 4°C with antibodies against PRMT6 (1:1000 dilution, Cell Signaling Technology, Danvers, MA, USA), cleaved PARP (1:1000; Cell Signaling Technology), β-actin (1:10,000; Santa Cruz Biotechnology, Santa Cruz, CA, USA), mono- and dimethyl arginine (1:500; Abcam, Cambridge, UK), p21 (1:1000, Cell Signaling Technology), p53(1:5000, Santa Cruz), histone H3 (1:50000, Abcam), H3R2me2a (1:500, Novus Biologicals, Littleton, CO, USA). Subsequently, the membranes were incubated with the secondary antibodies for 1 h at room temperature, and an Immobilon Western Chemiluminescent HRP Substrate (Millipore) was used for detection. The relative protein levels were calculated by comparison to the levels of β-actin.

### Quantitative real time PRC

RNA was extracted from cell lines using TRIzol® (Invitrogen, Carlsbad, CA, USA). High-capacity cDNA Reverse Transcription Kit from Applied Biosystems (Foster City, CA, USA) enabled first strand synthesis. PCR reactions were carried out using LightCycler 96 System (Roche Diagnostic) following the manufacture’s protocol. Primer sequences for human *GAPDH* (housekeeping gene), *PRMT6*, *p21* and *p53* are presented in Supplementary Table 1.

### Statistical analysis

Statistical analysis was performed using SPSS version 18.0 software (SPSS Inc., Chicago, IL, USA). In all patients, DFS was defined as the interval between the date of surgery and date of the first detection of recurrence or date of the last known follow-up without evidence of recurrence. OS was the time of the last visit for regular follow-up. Statistical analyses comparing the two groups were performed using *t*-tests, χ^2^ tests, or Fisher’s exact tests. Regression analysis was performed using the Cox proportional hazard regression model for both univariate and multivariate analyses. Survival rates were estimated using the Kaplan-Meier method and compared with the log-rank test. Statistical results were considered significant if *p* < 0.05.

## SUPPLEMENTARY MATERIALS FIGURES AND TABLES



## Abbreviations

PRMT: protein arginine methyltransferase
CRC: colorectal cancer
OS: overall survival
DFS: disease-free survival
FITC: fluorescein isothiocyanate
PI: propidium iodide.
