# Supplementary material for: The prognostic significance of protein arginine methyltransferase 6 expression in colon cancer
Source: Oncotarget. 2017 Dec 27;9(10):9010–20. doi: 10.18632/oncotarget.23809 (PMC5823663; doi:10.18632/oncotarget.23809)
Supplement: Supplementary file 1 [file oncotarget-09-9010-s001.pdf]

The prognostic significance of protein arginine methyltransferase 6 expression in colon cancer

SUPPLEMENTARY MATERIALS

A B

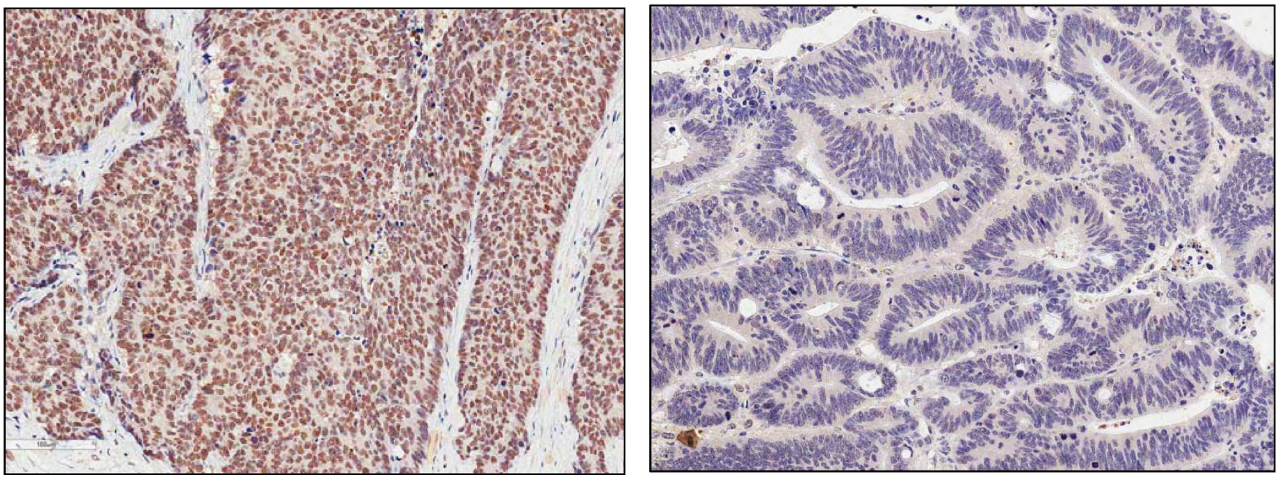

Supplementary Figure 1: Immunohistochemical PRMT6 staining CRC images. Images (´100) of PRMT6 positive (**A**) and PRMT6-negative tumors (**B**) from stage III CRC patients.

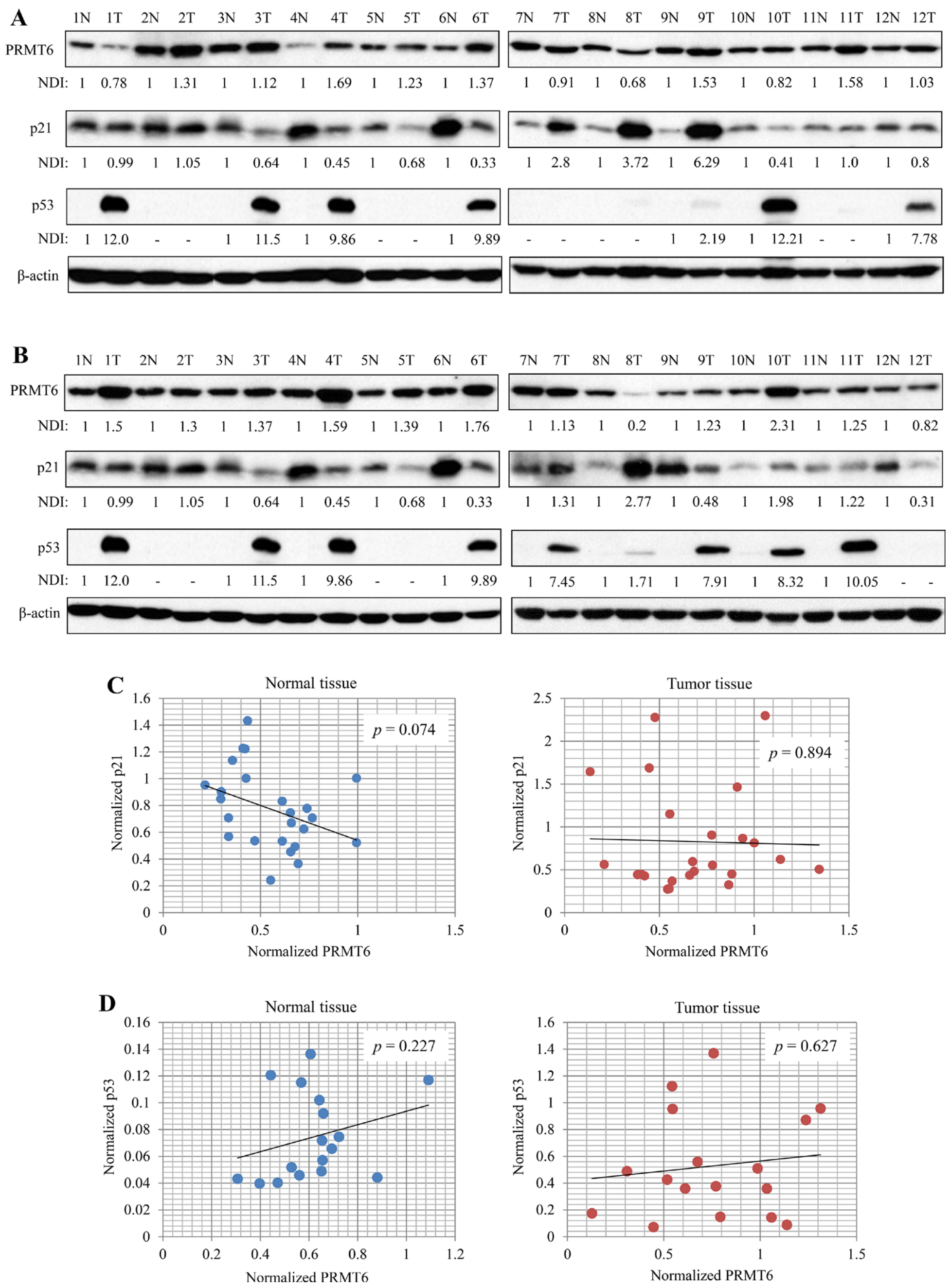

Supplementary Figure 2: Interrelationship between PRMT6 and tumor suppressors in 24 matched samples from the primary CRC and adjacent normal tissues paired from the same patient. Expressions of PRMT6, p21 and p53 in patients with stage II (**A**) and stage III (**B**) were examined by western blotting. Densitometric intensity of each protein was normalized to β-actin. NDI indicates normalized densitometric intensity. Densitometric values corresponding to each protein were plotted and interrelationships between PRMT6 and p21 (**C**) and p53 (**D**) analyzed.

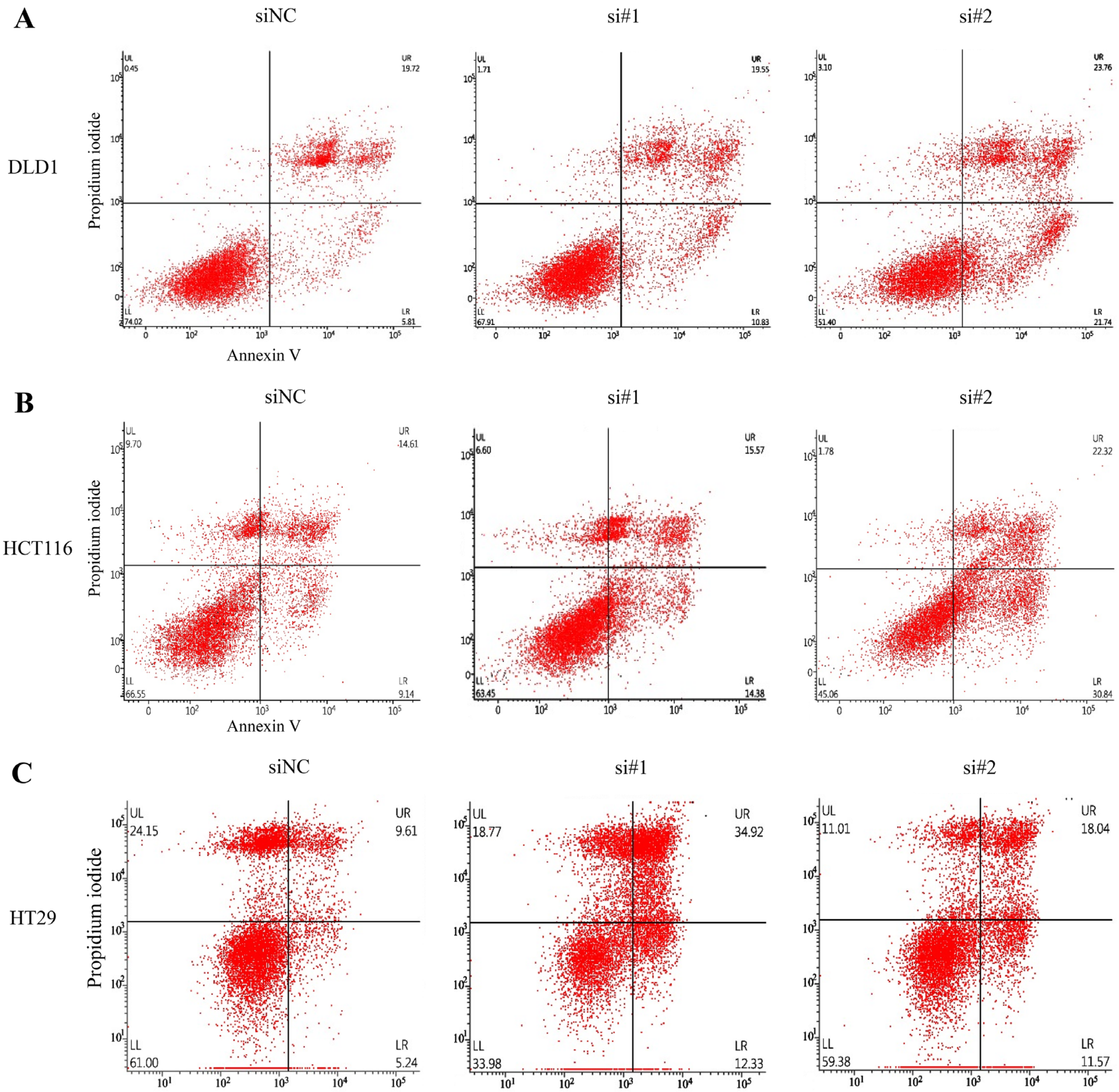

Supplementary Figure 3: Representative flow cytometry plots of siNC- and siPRMT6s-transfected CRC cells.

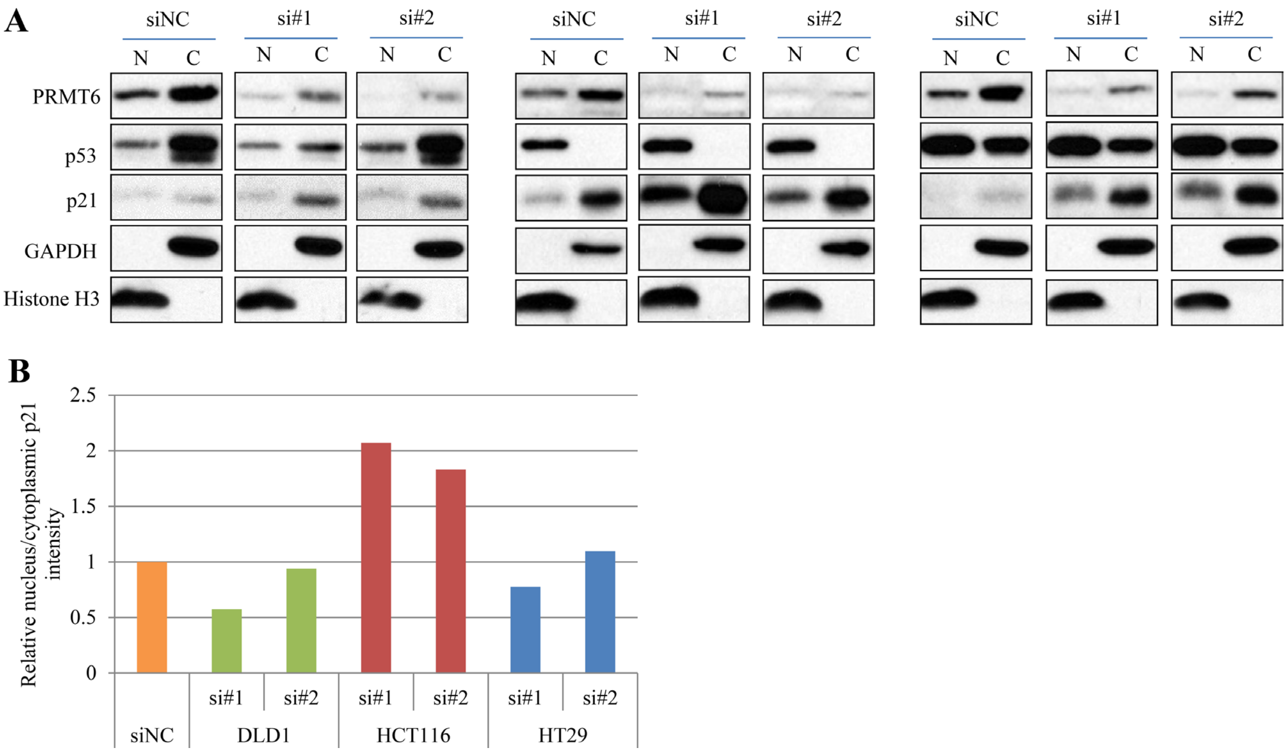

Supplementary Figure 4: Nuclear and cytoplasmic fractions of DLD1, HCT116 and DLD1 cells were prepared and analyzed by western blot. (A) The signal intensity of nuclear and cytoplasmic p21 was normalized by histone H3 signal and β-actin, respectively (B).

Supplementary Table 1: Sequences of primers used in the real-time PCR

Gene Name	Primer sequence	
PRMT6	Forward	5’-TGCTGCCACCTCGCCTTTTC-3’
	Reverse	5’-TTGGTCTTCTCCTCCTGGTCTCC-3
p21	Forward	5’-GGCAGACCAGCATGACAGATT-3’
	Reverse	5’-GCGGATTAGGGCTTCCTCTT-3’
p53	Forward	5’-AGACTGGGTCTCGCTTTGTTG-3’
	Reverse	5’-AGGCAAAGGCTGCAGTAAGC-3
GAPDH	Forward	5’-ATCATCCCTGCCTCTACTGG-3’
	Reverse	5’-CTGCTTCACCACCTTCTTGA-3

Supplementary Table 2: Patients' demographics

	Number (%) (Total *N* = 586)
**Gender *n* (%)**	
**Female**	221 (37.7)
**Male**	365 (62.3)
**Median age (range, years)**	59 (28–90)
**Location**	
**Colon**	436 (74.4)
**Rectum**	150 (25.6)
**Preoperative CEA (ng/ml) Median (range)**	1.93 (0.14-148.32)
**AJCC stage**	
**IIA**	269 (45.9)
**IIB**	29 (4.9)
**IIC**	10 (1.7)
**IIIA**	38 (6.5)
**IIIB**	240 (41.0)
**Tumor stage**	
**T1**	14 (2.4)
**T2**	26 (4.4)
**T3**	476 (81.2)
**T4**	70 (11.9)
**Nodal stage**	
**N0**	308 (52.6)
**N1**	216 (36.9)
**N2**	62 (10.6)
**Cell type**	
**Well-differentiated**	44 (7.5)
**Moderately-differentiated**	488 (83.3)
**Poorly-differentiated**	27 (4.6)
**Mucinous**	25 (4.3)
**Signet ring cell**	2 (0.3)
**Lymphatic invasion**	
**Negative**	430 (73.4)
**Positive**	156 (26.6)
**Vascular invasion**	
**Negative**	394 (67.2)
**Positive**	192 (32.8)
**Perineural invasion**	
**Negative**	411 (70.1)
**Positive**	175 (29.9)
**Adjuvant chemotherapy**	
**No**	45 (7.7)
**Yes**	458 (78.2)
**Undescribed**	83 (14.2)
**PRMT6 expression**	
**Negative**	447 (76.3)
**Positive**	139 (23.7)

Supplementary Table 3: Univariate and multivariate analyses for prognostic variables of disease-free and overall survival using Cox-proportional-hazards regression

(1) Disease-free survival

Predictors	Univariate Ratio of risk (95% CI)	*p*-value	Multivariate Ratio of risk (95% CI)	*p*-value
**Gender**		0.862		
**Female**	1.000			
**Male**	1.039 (0.672–1.607)			
**Age (years)**		0.107		
**< 60**	1.000			
**≥ 60**	0.697 (0.449–1.081)			
**Location**		< 0.001		0.006
**Colon**	1.000		1.000	
**Rectum**	2.175 (1.420–3.333)		1.851 (1.196–2.865)	
**CEA level**		0.001		0.076
**≤ 5 ng/ml**	1.000		1.000	
**> 5 ng/ml**	2.105 (1.330–3.332)		1.548 (0.956–2.505)	
**T stage**		0.174		
**T1–2**	1.000			
**T3–4**	2.223 (0.703–7.033)			
**N stage**		0.003		0.155
**N0**	1.000		1.000	
**N+**	1.944 (1.257–3.005)		1.448 (0.870–2.410)	
**Cell type**		0.689		
**WD/MD**	1.000			
**PD/MUC/SRC**	0.854 (0.394–1.849)			
**Lymphatic invasion**		< 0.001		0.094
**Negative**	1.000		1.000	
**Positive**	2.448 (1.604–3.735)		1.526 (0.930-2.503)	
**Vascular invasion**		< 0.001		0.003
**Negative**	1.000		1.000	
**Positive**	3.334 (2.126–5.229)		2.216 (1.318–3.725)	
**Perineural invasion**		0.010		0.195
**Negative**	1.000		1.000	
**Positive**	2.223 (1.208–4.088)		1.527 (0.805–2.897)	
**Adjuvant chemotherapy**		0.001		< 0.001
**Yes**	1.000		1.000	
**No/undescribed**	2.133 (1.359–3.349)		2.557 (1.602–4.080)	
**PRMT6 expression**		0.018		0.035
**Negative**	1.000		1.000	
**Positive**	1.711 (1.096–2.673)		1.630 (1.034–2.569)	

(2) Overall survival

Predictors	Univariate	*p*-value	Multivariate	*p*-value
	Ratio of risk (95% CI)		Ratio of risk (95% CI)	
**Gender**		0.441		
**Female**	1.000			
**Male**	1.295 (0.671–2.500)			
**Age (years)**		0.014		0.219
**≤ 60**	1.000		1.000	
**> 60**	2.191 (1.169–4.105)		1.509 (0.783–2.910)	
**Location**		0.124		
**Colon**	1.000			
**Rectum**	1.646 (0.872–3.107)			
**CEA level**		< 0.001		0.003
**≤ 5 ng/ml**	1.000		1.000	
**> 5 ng/ml**	3.482 (1.870-6.483)		2.654 (1.384–5.089)	
**T stage**		0.241		
**T1–2**	1.000			
**T3–4**	3.280 (0.451–23.863)			
**N stage**		0.014		0.282
**N0**	1.000		1.000	
**N+**	2.273 (1.177–4.388)		1.519 (0.710–3.254)	
**Cell type**		0.925		
**WD/MD**	1.000			
**PD/Muc/SRC**	1.051 (0.374–2.947)			
**Lymphatic invasion**		< 0.001		0.013
**Negative**	1.000		1.000	
**Positive**	3.732 (2.014–6.915)		2.389 (1.200–4.759)	
**Vascular invasion**		< 0.001		0.079
**Negative**	1.000		1.000	
**Positive**	3.230 (1.694–6.160)		1.895 (0.928–3.870)	
**Perineural invasion**		0.001		0.012
**Negative**	1.000		1.000	
**Positive**	3.676 (1.755–7.703)		2.694 (1.247–5.819)	
**Adjuvant chemotherapy**		< 0.001		<0.001
**Yes**	1.000		1.000	
**No/undescribed**	5.662 (3.061–10.476)		5.615 (2.914–10.819)	
**PRMT6 expression**		0.353		
**Negative**	1.000			
**Positive**	1.376 (0.702–2.696)			
